# Supplementary material for: Modular Chitosan-Based Adsorbents for Tunable Uptake of Sulfate from Water
Source: Int J Mol Sci. 2020 Sep 27;21(19):7130. doi: 10.3390/ijms21197130 (PMC7582897; doi:10.3390/ijms21197130)
Supplement: Supplementary file 1 [file ijms-21-07130-s001.pdf]

Supplementary Material

Modular Chitosan-Based Adsorbents for Tunable Uptake of Sulfate from Water

Bernd G. K. Steiger and Lee D. Wilson*
Department of Chemistry, University of Saskatchewan, 110 Science Place, Saskatoon, Saskatchewan S7N 5C9, Canada

*Corresponding Author: L. D. Wilson
e-mail: lee.wilson@usask.ca, Tel. +1-306-966-2961

Preliminary Adsorption Experiments

Preliminary results of sulfate adsorption (1000 ppm sodium sulfate solution) for non-modified and modified bead systems via cross-linking with glutaraldehyde and either no imbibing or subsequent doping with Fe(III) or calcium ions.

Table S1: Adsorption of inorganic sulfate on chitosan bead systems with and without cross-linking and cation imbibing.

Bead System	Q_e (mg/g)
No Modification	**<10**
CL-ratio 1:2 w/o imbibing	**<10**
CL-ratio 1:32 w/ calcium imbibing	**≈ 10**
CL-ratio 1:4 w/ Fe(III) imbibing	**55**
CL-ratio 1:4 w/ Ca imbibing	**42**

As expected, cross-linking alone does not lead to significant increase in sulfate uptake and remains below the detection limit of the chosen analytical method. However, a bead system with very little cross-linking but calcium imbibing indicated slight sulfate adsorption around the detection limit. With increased cross-linking (CL-ratio 1:4) and imbibing two different metals, the uptake performance in comparison to the purely cross-linked material (CL-ratio 1:2) increased measurably in both cases regardless of the nature of the cation. This suggested that calcium, although not as good as iron(III), is a suitable agent for modification and metal retention on the surface is the key component in designing tunable bead systems for sulfate uptake.

Relative number of articles concerned with sulfate, phosphate, nitrate

Figure S1 shows the number of articles that address the adsorption-based removal of sulfate.

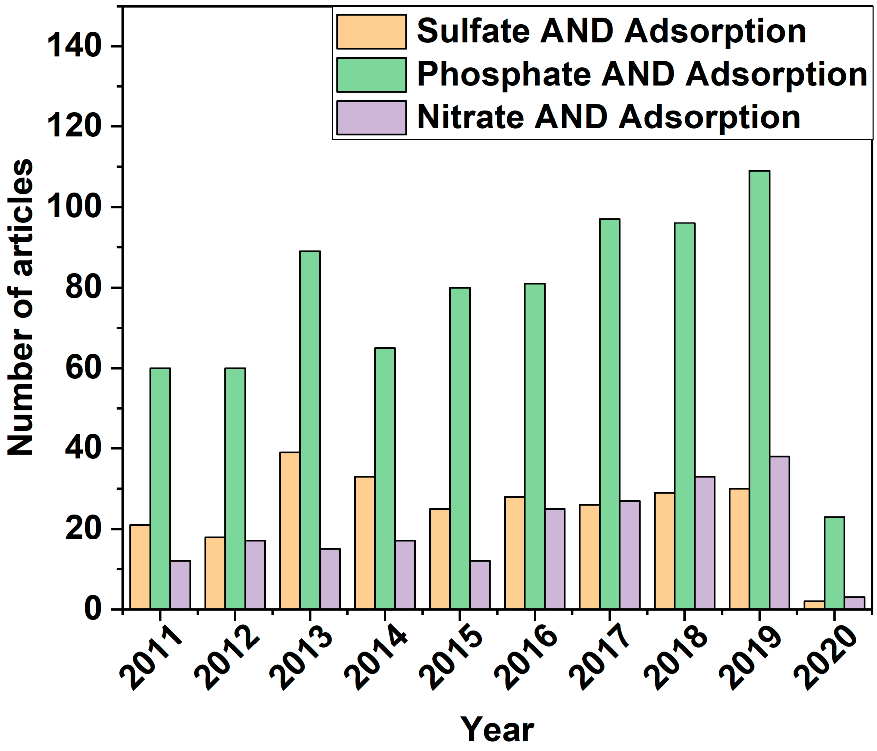

Figure S1: Number of articles published between 2011 and early 2020 with the key words "sulfate" and "adsorption", "phosphate" and "adsorption" and "nitrate" and "adsorption" in the article title (source: Scopus).

X-Ray Photoelectron Spectroscopy

Surface characterization of the synthesized bead systems played was crucial in developing an understanding of the materials and XPS was used to analyze the effect of cross-linking and metal imbibing. The XPS analysis (wide scan) showed presence of calcium within the unmodified system, most likely impurities from the purchased chitosan used during synthesis as seen in Fig. S2.

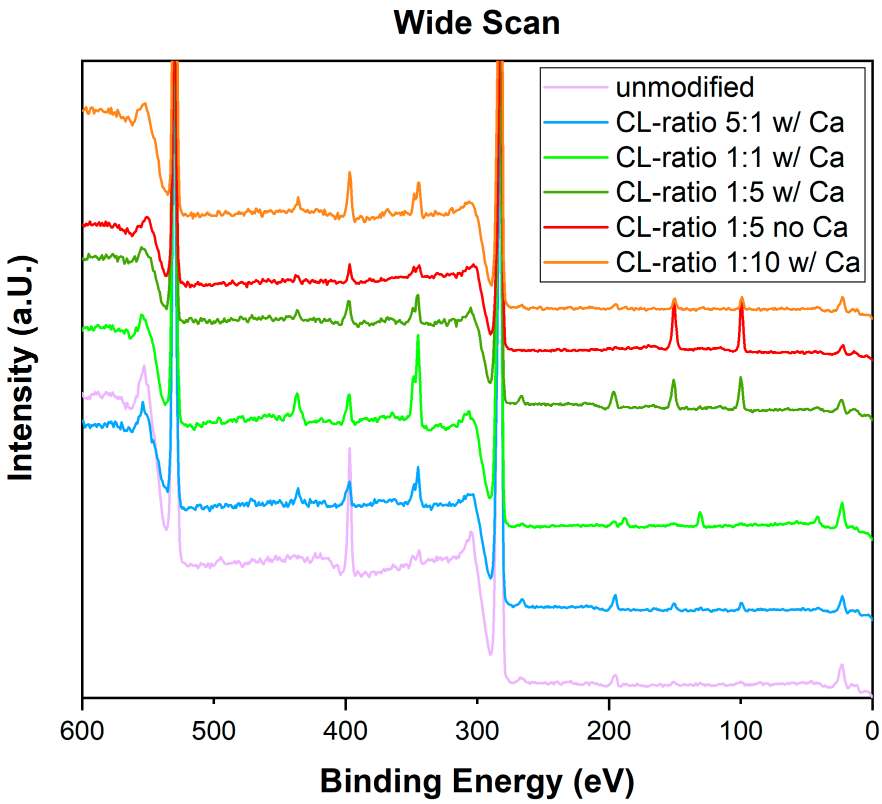

Figure S2: XPS analysis (wide scan) of bead systems with different cross-linking ratios of glutaraldehyde to chitosan monomers and calcium imbibing.

The narrow scan of the nitrogen region showed increased presence of a side band after modification rather modification independently of calcium imbibing suggesting no strong coordination effects of calcium ions (*cf.* Fig. S3). The narrow scan of calcium (*cf.* Fig. S4) on the other hand shows no peak shift itself suggesting no strong chelation or change in coordination. The narrow scan of carbon highlighted a unique separate band of the material with the lowest cross-linking ratio of 1:10 (283.85 eV) which more and more developed into a smaller shoulder peak of 282.2 eV with decreasing intensity. The same trend was observed with 285.4 eV with the exception of the material with a CL-ratio of 1:1 w/ Ca which showed a separate peak at 286.2 eV that is not observed for the other materials (*cf.* Fig. S5).

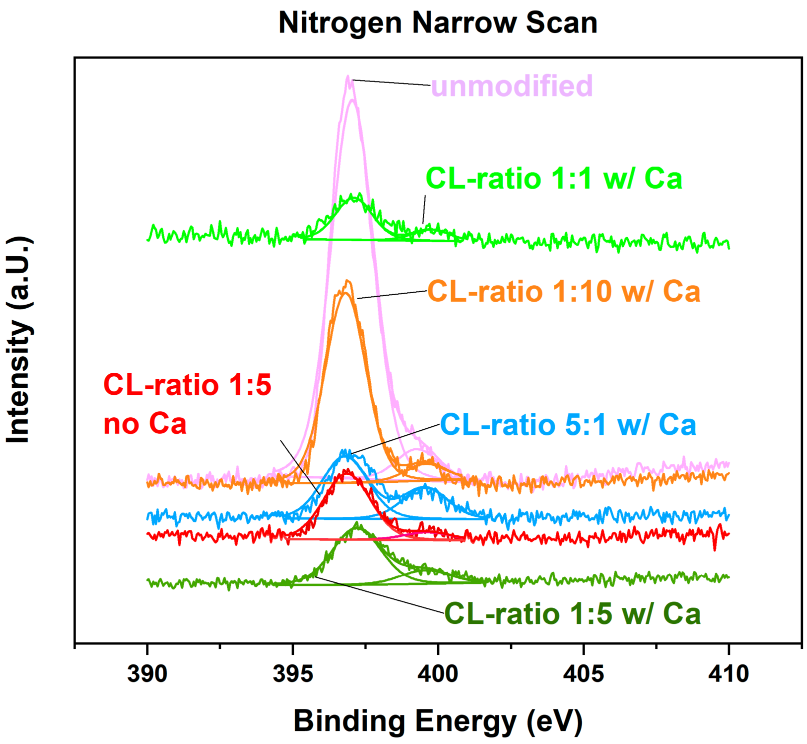

Figure S3: XPS analysis (narrow scan) of the oxidation state of nitrogen in bead systems with variable cross-linking ratios of glutaraldehyde to chitosan monomers and with calcium imbibing.

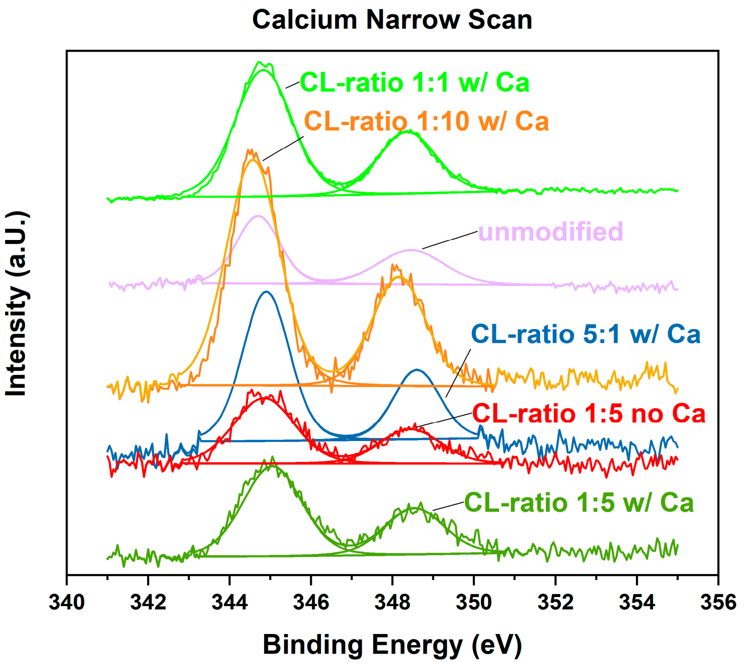

Figure S4: XPS analysis (narrow scan) of the oxidation state of calcium in bead systems with different cross-linking ratios of glutaraldehyde to chitosan monomers after calcium imbibing.

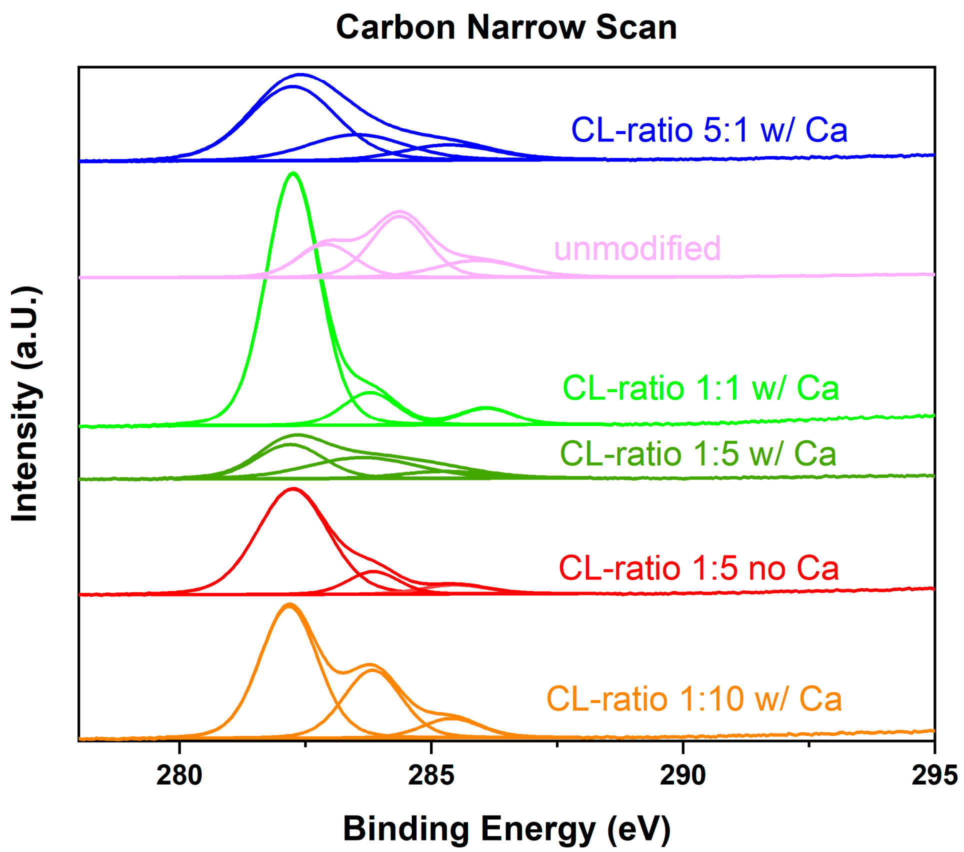

Figure S5: XPS analysis (narrow scan) of the oxidation state of carbon in bead systems with different cross-linking ratios of glutaraldehyde to chitosan monomers after calcium imbibing.

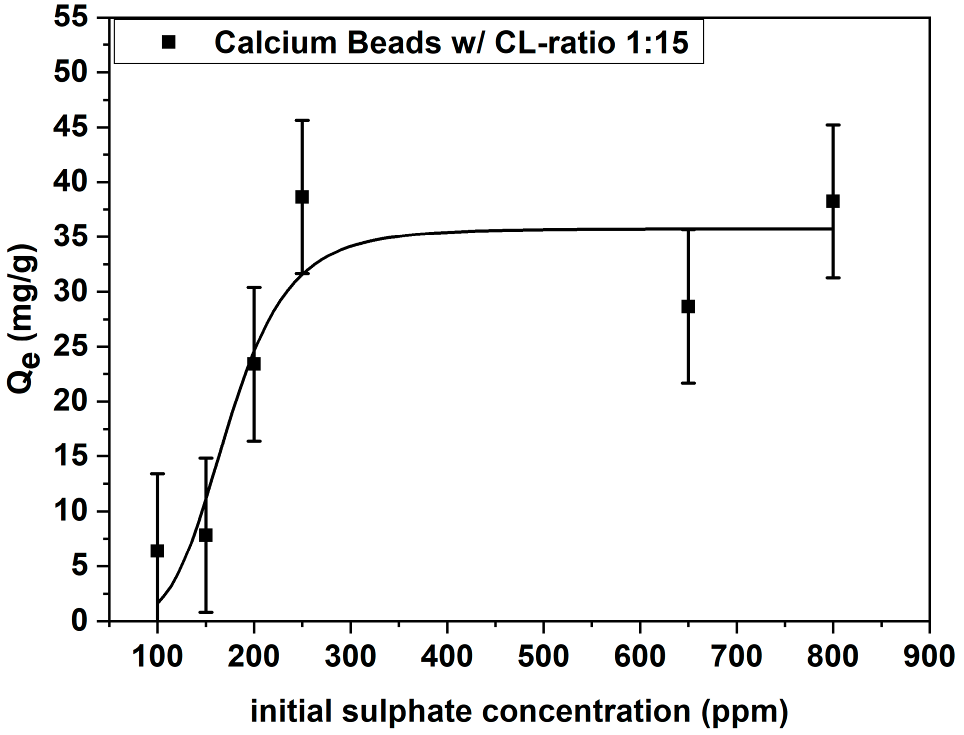

Figure S6: Sulfate adsorption isotherm with cross-linked and calcium imbibed beads (CL-ratio 1:15) at pH 5 abd 295 K. The solid line is the best-fit to the Langmuir-Freundlich isotherm model.

Isotherm and Kinetic Parameters

Analysis of the values obained via isotherm studies may allow for more insights into binding of adsorbate to adsorbent. The obtained isotherm values from the Sips istherm analysis are provided in Table S2.

Table S2: Determination of Q_e, k and n values of four selected, calcium imbibed bead systems.

Bead System	*Q_e (mg/g)*	*k*	*n*
1:10 (CL-ratio, w /Ca	62 ± 7.8	$7x10^{-6}$ ± $3.0x10^{-5}$	2.2 ± 0.8
1:5 (CL-ratio, w /Ca	50 ± 2.0	$6x10^{-7}$ ± $3.1x10^{-6}$	3.0 ± 1.0
1:1 (CL-ratio, w /Ca	107 ± 15.5	$1x10^{-8}$ ± $1.2x10^{-7}$	3.3 ± 2.0
5:1 (CL-ratio, w /Ca	140 ± 10.3	$1x10^{-12}$ ± $3.6x10^{-11}$	4.9 ± 6.1

Kinetics measurement of calcium imbibed bead systems at 2000 ppm sulfate concentration were undertaken with ca. 500mg beads (CL-ratio 5:1)

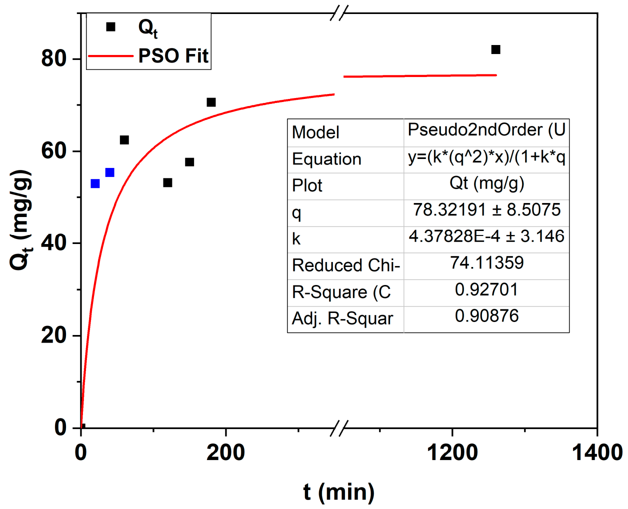

Figure S7: Kinetics of beads (CL-ratio 5:1 w/ Ca) at 2000 ppm sulfate concentration and ca. 500mg beads.

Size Distribution

To evaluate the size distribution of the bead systems, the diameter of multiple beads (n=15) per system was measured via a digital caliper (see Fig. S8 and Table S3).

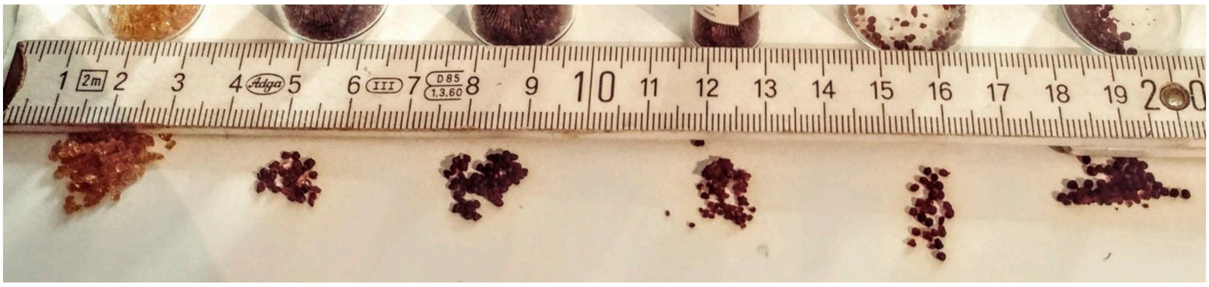

Figure S8: Size of different bead systems from left to right: non-modified, 1:15 Cl-ratio, 1:10 Cl-ratio, 1:5 Cl-ratio, 1:1 Cl-ratio and 5:1 Cl-ratio.

Table S3: Bead size estimates* in the dry state.

**Bead System**	**Mean** (mm)	**Median** (mm)	**Standard Dev.** (mm)	**Spread** (mm)
Non-modified	1.51	1.46	0.17	0.60
1:15 Cl-ratio	1.34	1.32	0.20	0.81
1:10 Cl-ratio	1.28	1.29	0.18	0.64
1:5 Cl-ratio	0.96	0.93	0.14	0.50
1:1 Cl-ratio	1.42	1.43	0.15	0.56
5:1 Cl-ratio	1.48	1.49	0.17	0.66

* The mean size was obtained using a digital caliper over 15 individual measurements
